# FilterDCA: Interpretable supervised contact prediction using inter-domain coevolution

**DOI:** 10.1371/journal.pcbi.1007621

**Published:** 2020-10-09

**Authors:** Maureen Muscat, Giancarlo Croce, Edoardo Sarti, Martin Weigt

**Affiliations:** Sorbonne Université, CNRS, Institut de Biologie Paris Seine, Biologie Computationnelle et Quantitative – LCQB, 75005 Paris, France; Max Planck Institute for Biophysical Chemistry, GERMANY

## Abstract

Predicting three-dimensional protein structure and assembling protein complexes using sequence information belongs to the most prominent tasks in computational biology. Recently substantial progress has been obtained in the case of single proteins using a combination of unsupervised coevolutionary sequence analysis with structurally supervised deep learning. While reaching impressive accuracies in predicting residue-residue contacts, deep learning has a number of disadvantages. The need for large structural training sets limits the applicability to multi-protein complexes; and their deep architecture makes the interpretability of the convolutional neural networks intrinsically hard. Here we introduce FilterDCA, a simpler supervised predictor for inter-domain and inter-protein contacts. It is based on the fact that contact maps of proteins show typical contact patterns, which results from secondary structure and are reflected by patterns in coevolutionary analysis. We explicitly integrate averaged contacts patterns with coevolutionary scores derived by Direct Coupling Analysis, improving performance over standard coevolutionary analysis, while remaining fully transparent and interpretable. The FilterDCA code is available at http://gitlab.lcqb.upmc.fr/muscat/FilterDCA.

## Author summary

The *de novo* prediction of tertiary and quaternary protein structures has recently seen important advances, by combining unsupervised, purely sequence-based coevolutionary analyses with structure-based supervision using deep learning for contact-map prediction. While showing impressive performance, deep-learning methods require large training sets and pose severe obstacles for their interpretability. Here we construct a simple, transparent and therefore fully interpretable inter-domain contact predictor, which uses the results of coevolutionary Direct Coupling Analysis in combination with explicitly constructed filters reflecting typical contact patterns in a training set of known protein structures, and which improves the accuracy of predicted contacts significantly. Our approach thereby sheds light on the question how contact information is encoded in coevolutionary signals.

## Introduction

The prediction of protein structure using amino-acid sequence information has seen important progress in the last decade. This progress is substantially based on two subsequent methodological advances: unsupervised sequence-based coevolutionary models, and subsequent structure-based supervision using deep learning.

First, global coevolutionary methods like Direct Coupling Analysis (DCA) [1, 2], PSICOV [3] or Gremlin [4, 5] have allowed to identify direct coevolutionary couplings by modeling the sequence variability found in sufficiently large homologous protein families. It was found that the largest coevolutionary couplings correspond, with high probability, to residue-residue contacts inside or between proteins [6, 7]. The potential for tertiary and quaternary protein structure prediction is evident, and many interesting cases have been published [8–11]. However, the fully unsupervised character of coevolutionary models is limiting their wide-scale applicability: they are based only on statistical models of sequence ensembles, and do not make use of available structural information. This fact leads, in turn, to a restricted number of accurately predicted contacts, in particular in the case of protein families containing only a limited number of sequences.

In the case of tertiary structure, this problem has been solved more recently. Benefitting from the large numbers of proteins with experimentally determined structures available in the PDB, and from the large amount of residue-residue contacts (and non-contacts) in each structure, supervised machine-learning techniques have allowed to substantially increase the accuracy in predicting residue-residue contacts and even distances. In particular convolutional neural networks (CNN) based on deep learning have shown success, cf. the performance of methods like RaptorX [12], DeepMetaPSICOV [13] and AlphaFold [14] in the last editions of the CASP structure prediction competition [15].

Despite their impressive predictive performance, CNN and other deep neural architectures have some important disadvantages. First, they need to be trained on large datasets. While these are available for tertiary protein structures, the applicability to inter-protein contact prediction and protein-complex assembly has remained limited, even if promising results have been found when training was performed on intra-protein contacts, but testing on inter-protein contacts, resulting in a RaptorX variant called RaptorX-ComplexContact [16]. Second, deep learning is intrinsically hard to interpret. It remains normally unclear how deep networks extract information from data, and how they use it to annotate residue pairs as contacts or non-contacts.

Our works aims at assessing the following question: Can we understand how contact information is encoded in the patterns of coevolution unveiled by DCA-type unsupervised methods? Can we use this to improve on DCA in a simple and interpretable way? It is very unlikely that performances similar to the best CNN-based predictors can be achieved, but a better understanding of coevolutionary patterns may be exploited in future by a next generation of deep neural networks.

The architecture of CNN may actually give a first hint. Instead of looking individually to residue pairs, their first layer applies convolutional filters to a neighbourhood of each residue pair. Protein contact maps are not random graphs, they are locally structured, and this structure can be exploited for contact prediction. As was pointed out in [17], the density of contacts is higher in the neighbourhood of a contact than in the neighbourhood of a non-contact.

Based on this observation, our questions may be made more precise: can we use local contact patterns to construct simple and interpretable supervised approaches to contact prediction? Can we use such approaches in the case of inter-protein and inter-domain contacts, which are less represented in the protein data bank PDB [18] than intra-domain contacts and thus provide less training data?

To this end, we introduce FilterDCA: we first analyse contact patterns related to different secondary structure elements, and show that these are faithfully reflected by the scores derived using plmDCA (i.e. DCA based on pseudo-likelihood maximisation) [19]. We therefore use average contact patterns as explicit filters for the DCA predictions, and combine them with the standard DCA score using simple logistic regression. While our learning and testing procedure uses domain-domain interactions inside multi-domain proteins, due to their better availability in the PDB, we show that the prediction of inter-protein contacts is significantly improved, too. We think that these results are interesting, because they allow to take a step forward in understanding how contact information is actually hidden in sequence alignments.

A next logical step (going beyond the scopes of this work) would be to understand if this kind of information is also used by CNN-based contact predictors, or if their architecture can be refined to better take this information into account. Surprisingly, FilterDCA reaches perfomances comparable to the CNN-based contact predictor PconsC4 [20], a further development of the beforementioned approach of [17]. When applied to protein-protein interactions, its performance remains, however, behind two of the currently best-performing CNN-based predictors: RaptorC-ComplexContact [16] and DeepMetaPSICOV [13].

The plan of the paper follows this reasoning: We first find characteric patterns, which are shared by protein contact maps and coevolutionary couplings. Using domain-domain interactions as a proxy for protein-protein interactions, we develop FilterDCA as a simple supervised contact predictor combining these contact patterns with the outcome of standard DCA, leading to a cleaning of predicted contact maps and thereby to higher prediction accuracy. Subsequently, we show that results can be extended to protein-protein interactions, and provide a comparison with the performance of deep-learning based contact predictors.

## Results and discussion

To improve DCA-based contact prediction between interacting domains, we have collected an extensive dataset of more than 2500 intra-protein domain-domain interactions of experimentally known protein structures, containing more than 6 ⋅ 10^5^ inter-domain contacts, cf. *Materials and Methods*. For each of these domain pairs, a multiple-sequence alignment (MSA) is created, with each line containing the aligned sequences of both interacting domains. According to the effective number *M*_eff_ of sequences (as calculated by plmDCA in standard settings), we distinguish three cases: large (*M*_eff_ > 200), medium (50 < *M*_eff_ < 200), and small (*M*_eff_ < 50) MSAs. These data will be used throughout the paper, if not explicitly stated differently.

### DCA scores reflect typical inter-domain contact patterns

While DCA uses rather sophisticated approaches for global statistical modeling of MSAs of families of homologous proteins, the subsequent residue-residue contact prediction is surprisingly naïve: coevolutionary coupling matrices *J*_*ij*_(*a*, *b*) of dimension 21 × 21 between two residue positions *i* and *j* (MSA columns) are compressed into a single ad-hoc score, either using direct information *DI*_*ij*_ [1] or, more frequently, by calculating a statistically corrected Frobenius norm *F*_*ij*_ [19], and the largest scores are interpreted as potential contacts. As is shown in *Materials and Methods*, this strategy is informative about contacts in the case of large MSAs, becomes questionable in the case of intermediate MSAs, and totally loses signal for small MSAs.

This strategy might anyway be optimal if contact maps were totally random. However, contact maps show important patterns, mainly based on secondary structures: *β*-sheets are characterised by diagonal patterns, *α*-helices give rise to almost periodic patterns with a period of 3-4 corresponding to one helical turn: when a pair (*i*, *j*) is in contact, the residues *i* ± 2 (resp. *j* ± 2) are on the opposite face of the helix, and thus probably do not form contacts with any residue close to *j* (resp. *i*), while *i* ± 3, 4 (resp. *j* ± 3, 4) are again on the same face as *i* (resp. *j*).

To exploit such patterns, we have classified all residues according to their secondary structure into three classes: helical (H), extended (E) and other (O). Each inter-domain contact can now be classified by the secondary-structure annotation of the two contacting residues. Here we present results exclusively for the classes HH of two helical residues in contact, and EE for two contacting residues which are both located in *β*-strands, which show very characteristic contact patterns. All other classes are included in the contact prediction, too, but due to the absence of clear contact patterns, they will not particularly benefit from our new approach, as compared to standard DCA.

**Fig 1:**
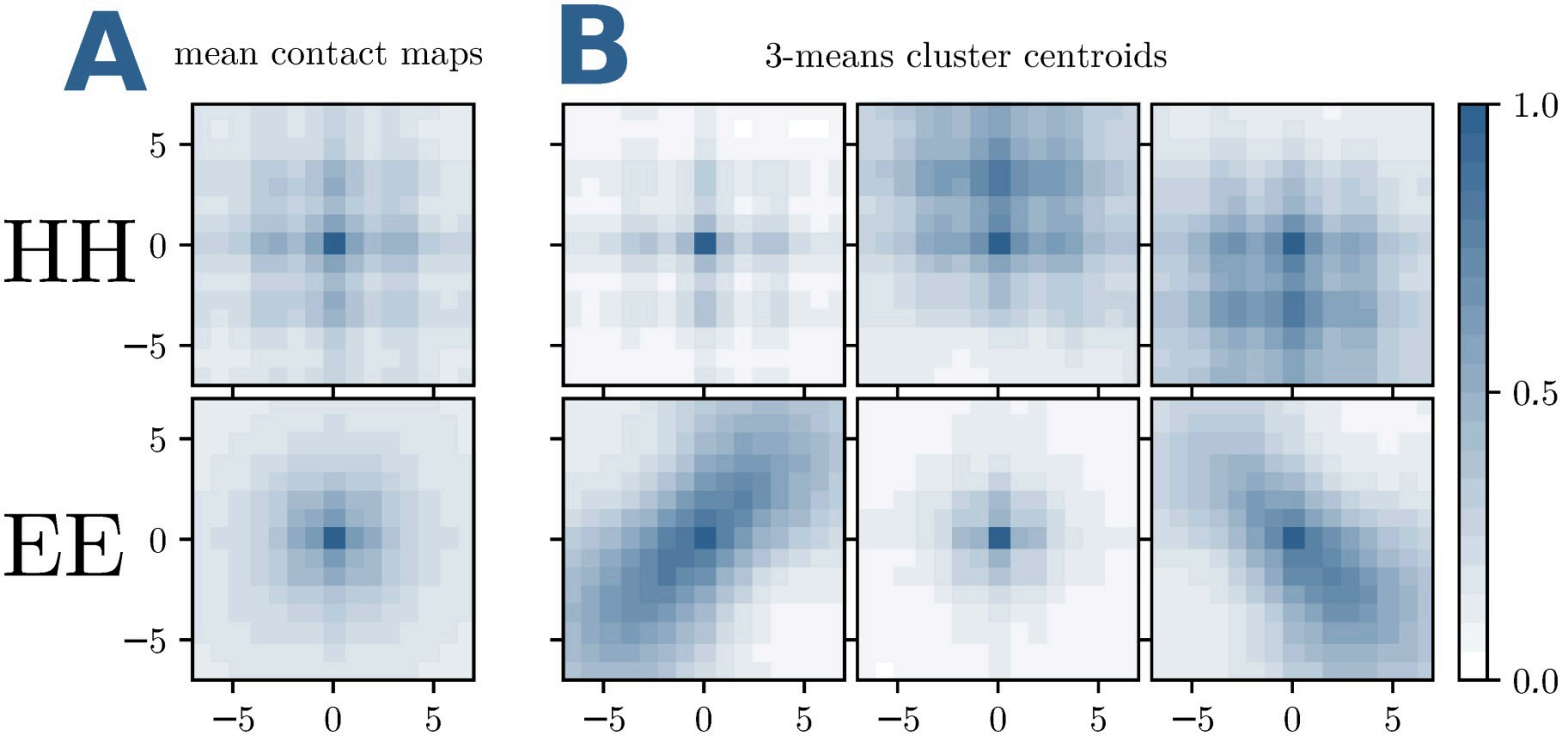
Typical patterns in inter-domain contact matrices. (A) The frequencies of contacts in a 15×15 contact-map window around an HH or EE contact are displayed. The average is done over the 46978 HH and 12281 EE contacts of the training set. The mean contact matrices are a combination of parallel, anti-parallel or mixed HH and EE contacts. (B) We disentangle them with a *k*-means clustering with *k* = 3. The 6 resulting centroids, 3 for a central HH inter-domain contact (upper row) and 3 for an EE contact (lower row), show pronounced patterns and can be used to filter DCA predictions.

For these two groups, HH and EE contacts, we calculate average contact maps over windows centered in all contacts (*i*, *j*), which are realised in the above-mentioned inter-domain interaction dataset for large families, cf. *Materials and Methods*. For a window size of 15×15, we show the results in Fig 1A; the entries give the estimated probabilities to find another contact in the corresponding position relative to the given contact (*i*, *j*) in the center of the window. While the case of HH contacts shows a clear pattern with the expected periodicity of 3-4 positions, i.e. in coherence with one turn of the *α*-helices, the case of EE contacts shows a non-informative pattern, where the contact density decays simply with the distance from the central EE contact.

The picture changes when we refine the analysis using *k*-means clustering with *k* = 3 of the HH and EE contact matrices. The HH patterns remain similar, unveiling some fine structure in the HH contact ensemble. The EE case now becomes highly informative, we clearly observe the two diagonal patterns corresponding to parallel resp. anti-parallel *β*-strands. The third cluster assembles all other cases, like crossing *β*-strands.

**Fig 2:**
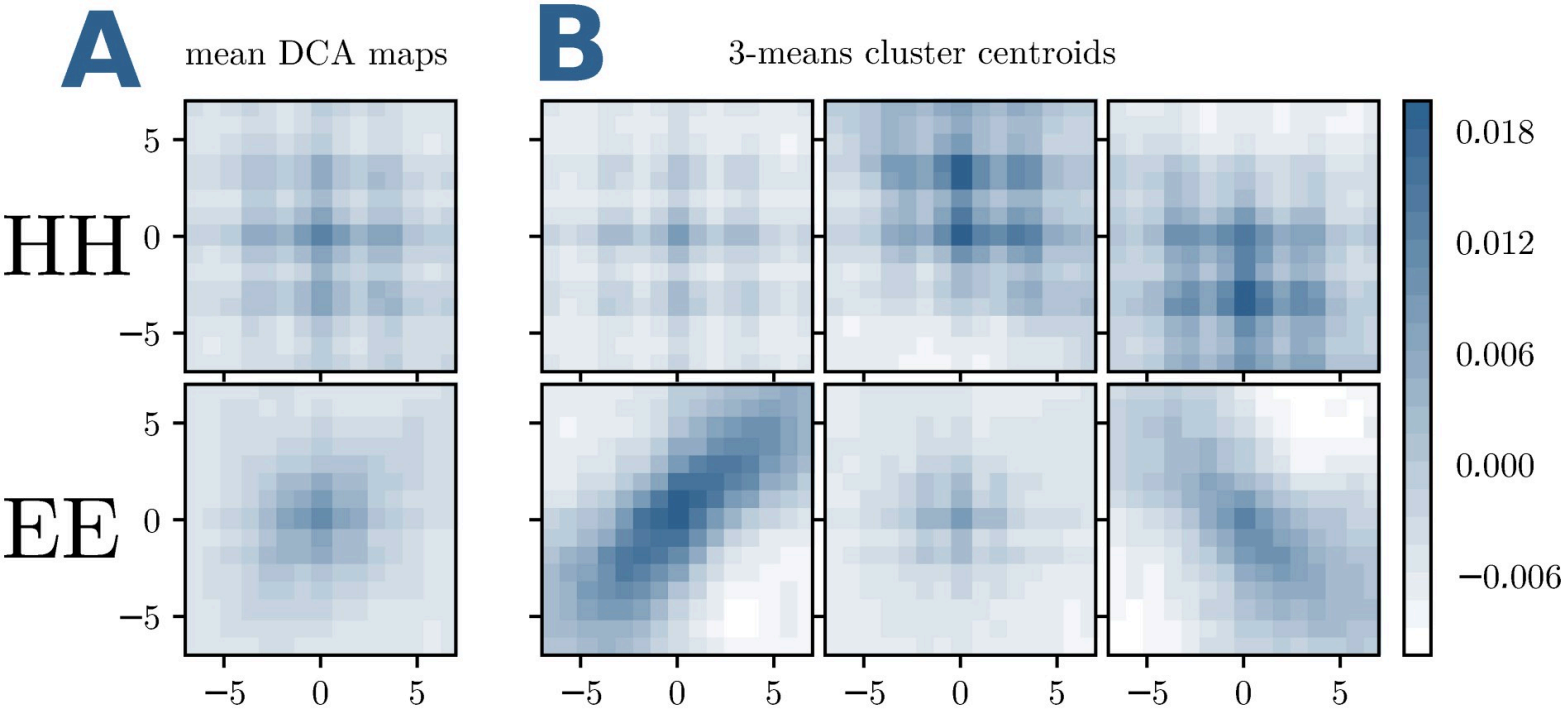
Typical patterns in DCA-score matrices. (A,B) The average DCA scores in a 15 × 15 window around an HH or EE contact are displayed, using the same selection of contacts as in Fig 1 for panels A, and the same sub-clustering for panels B.

Are these contact patterns reflected by the matrices of DCA scores? To answer this question, we calculate average DCA-scores for windows centered around exactly the same contacts as those used in Fig 1. Doing so, we average out site specificities and noise, and only coherent local patterns remain visible. As becomes evident in Fig 2, the resulting patterns have the same structure as the averaged contact maps. However, it becomes also evident that the average DCA scores are very small, as compared to DCA scores, which are indicative for contacts, cf. *Materials and methods*.

This double observation is the starting point for our supervised contact predictor in FilterDCA: while many contacts have small DCA scores, i.e. they would not be predicted as contacts in standard plmDCA, they can still have a characteristic DCA pattern in their neighbourhood, and thus potentially be identified as contacts. On the other hand, a strong DCA score will remain an important indicator for a contact to exist.

### Integrated contact patterns and DCA improve inter-domain contact prediction

**Fig 3:**
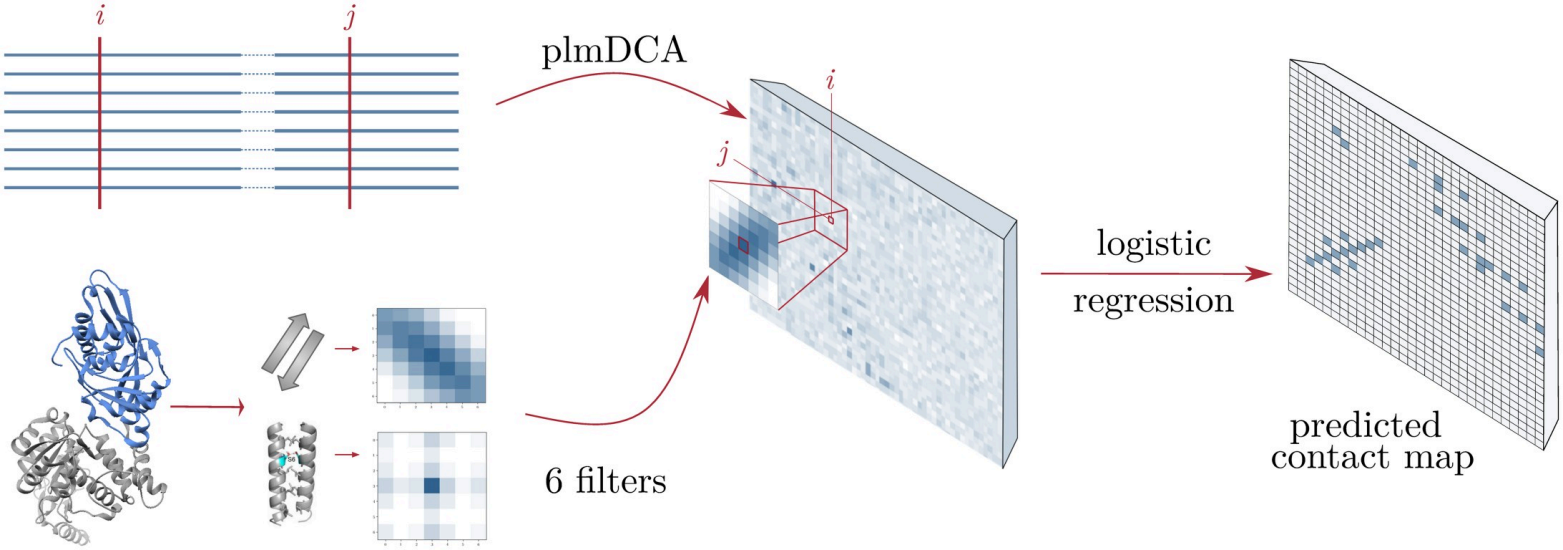
General scheme of FilterDCA: Our approach combines the results of plmDCA applied to two-domain MSAs with structural filters constructed as average contact matrices using 6 contact classes. Structural supervision is used to learn a logistic regression based on the plmDCA score itself, and the best correlation with one of the six structural filters.

To this aim, we have developed a simple and transparent strategy to integrate plmDCA with the structural filters to improve inter-domain contact prediction, cf. Fig 3. The idea is to integrate the standard plmDCA score, which is known to be a good contact predictor when assuming large enough values, with a coherence measure between our contact filters and the corresponding window of plmDCA scores.

To this end, we define two informative features **x** = (*x*_1_, *x*_2_) for each pair of residues (*i*, *j*), as a function of the DCA scores *F*_*ij*_ and the structural filters

$$\begin{array}{ccc} x_{1} & = & F_{ij}\\ x_{2} & = & \underset{\text{filters}\;f}{\text{max}}\rho \left(f,D_{ij}\right) \end{array}$$ (1)

with *D*_*ij*_ being a window of DCA scores around residue pair (*i*, *j*), and *f* denoting the 6 filters (which, to exclude overlaps between training and test data, are now determined using the dataset of small MSAs, cf. *Material and Methods*), and *ρ* being the Pearson correlation. Note that in both *D*_*ij*_ and *f* the central element has been removed, since it would introduce a strong redundancy with the first feature. The size of the window is a free parameter whose influence will be examined. In the following, we will refer to *x*_1_ as “DCA score”, and to *x*_2_ as “filter score”.

To integrate these two features into a single contact predictor, we use simple logistic regression. The probabilities to belong to the class *contact* (⊕) or *non-contact* (⊖) are thus given by:

$$\begin{array}{ccc} P(⊕|x) & = & \frac{e^{\mathrm{wx}+w_{0}}}{1+e^{\mathrm{wx}+w_{0}}}\\ P(⊖|x) & = & \frac{1}{1+e^{\mathrm{wx}+w_{0}}} \end{array}$$ (2)

where the bias *w*_0_ and the weights **w** = (*w*_1_, *w*_2_) are optimised using a training set of 50% of the data. This is done independently for the large and intermediate MSAs, cf. *Materials and Methods* for the details of the implementation.

**Fig 4:**
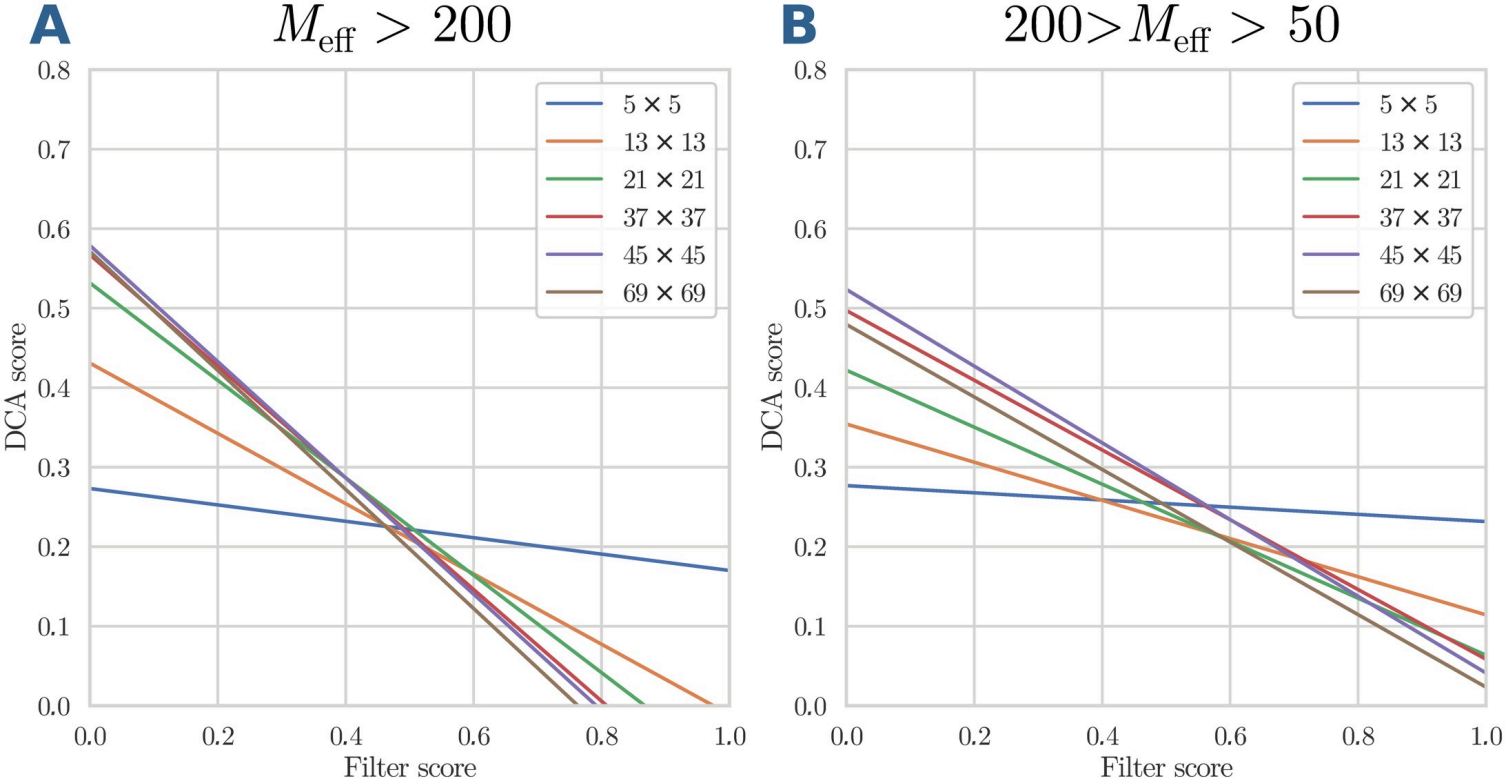
Decision boundary for logistic regression. The lines show, for large (panel A) and medium (panel B) MSA sizes, the decision boundary defined by *P*(⊕|**x**) = 1/2, for different filter sizes raging from 5 × 5 to 69 × 69.

A first insight into the relative importance of the two features—the standard DCA score and the local coherence with the typical contact patterns measured by the filter score—can be gained from Fig 4. For small filter sizes, the decision boundary is almost horizontal, i.e. the decision is almost exclusively determined by the DCA score, and the filter score has little influence. The decision boundary is located mainly between 0.2 and 0.3, in perfect agreement with the crossing points of the histograms of contacts and non-contacts, cf. *Materials and methods*.

This changes when larger windows are used as filters. The decision boundary becomes tilted. Not only pairs (*i*, *j*) of smaller DCA score can be predicted to be contacts when being in an environment of high filter score, but also relatively large DCA scores may be discarded when not being related to a reasonably large filter score. Coherence of the DCA signal around a residue pair with local contact patterns thus has the potential to discover otherwise discarded contacts, and to prune large DCA scores judged to be isolated noise due to an incoherent environment.

**Fig 5:**
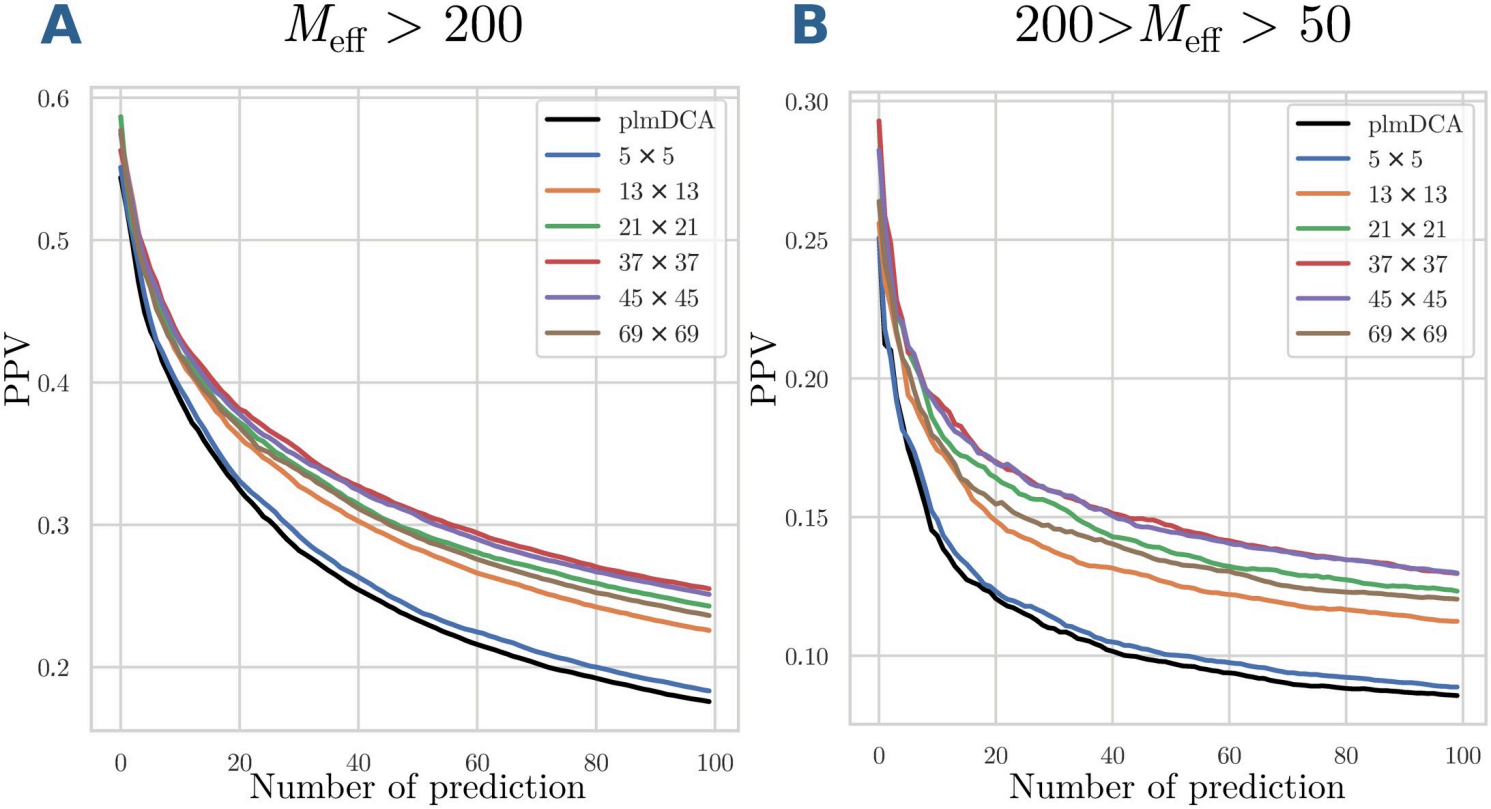
Positive predictive values of FilterDCA for inter-domain contact prediction. PPV are shown as a function of the number of predictions for large (panel A) and medium (panel B) MSA sizes, averaged over the different domain-domain interactions in the test set. Different filter sizes are compared to standard plmDCA.

Is this potential actually realised and leads to better contact predictions in the test set of protein families, which were not used for model learning? Fig 5 shows the positive predictive value (PPV = TP/(TP+FP)) as a function of the number of predictions (TP+FP), averaged over all families in the test sets for large and intermediate MSA sizes. A comparison with standard plmDCA shows that the application of structural filters effectively improves the inter-domain contact prediction. However, the quality of the first predicted contacts does not change a lot, i.e. it is still dominated by the quality of the plmDCA prediction. On the contrary, the decay of the PPV with the number of predicted contacts is substantially slower. The maximal PPV is reached for quite large filters (size 45 × 45), but then it decreases again, as testified by the curve for 69 × 69 filters. Intuitively we thus find that FilterDCA is able to help only if DCA alone finds some contact signal. Coherence with contact patterns over quite large environments of the considered residue pairs (*i*, *j*) is most informative, but even larger filters lead to a decay since the filters take into account too distant and thus too structurally-variable regions.

**Fig 6:**
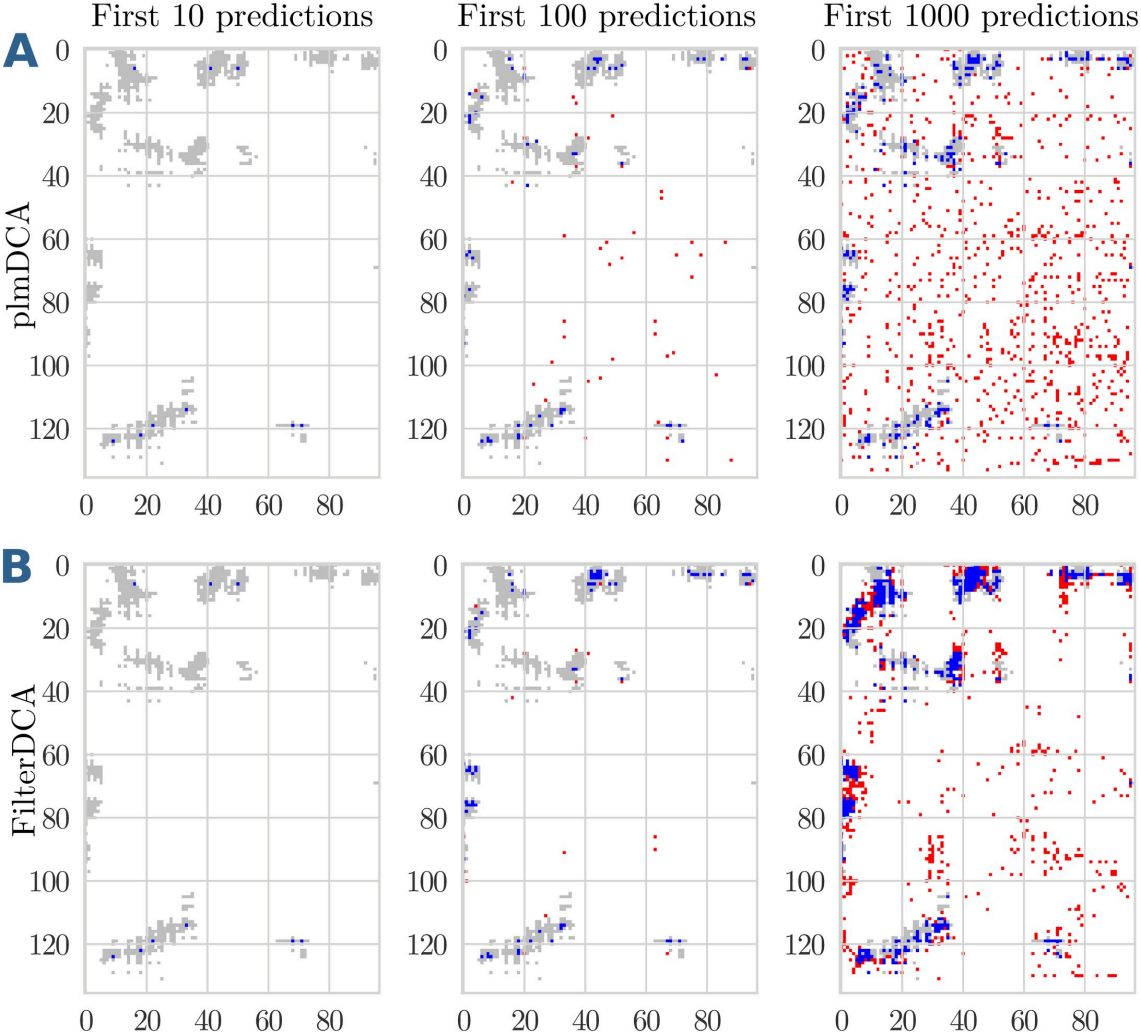
Contact map prediction for the interaction between the domain families PF02773 and PF00438 co-occurring in S-adenosylmethionine synthetases. The upper panels (A) show the predictions of plmDCA, the lower panels (B) those of FilterDCA, for the first 10, 100 or 1000 predicted contacts. In each figure, the native contacts are shown in grey, TP predictions of the two methods in blue, FP predictions in red. This example shows a case where plmDCA detects some signal, which is subsequently cleaned by FilterDCA.

The observed behaviour—an initial PPV similar to plmDCA, but a slower decay of the PPV—can be understood when looking in more detail into single domain-domain interactions. Fig 6 show a favourable case. A comparison of plmDCA (Panels A) and FilterDCA (Panels B) shows that the very first predictions are almost identical, and consequently have close to identical PPV. However, when more predictions are included, the two algorithms behave in a very different way. While the predictions of plmDCA are more distributed over the plane, and therefore predict many isolated pairs as contacts, FilterDCA predicts more compact clusters of contacts, in closer vicinity to the first predicted contacts, and new clusters appearing when more and more predictions are added. Most isolated FP are thereby successfully removed.

**Fig 7:**
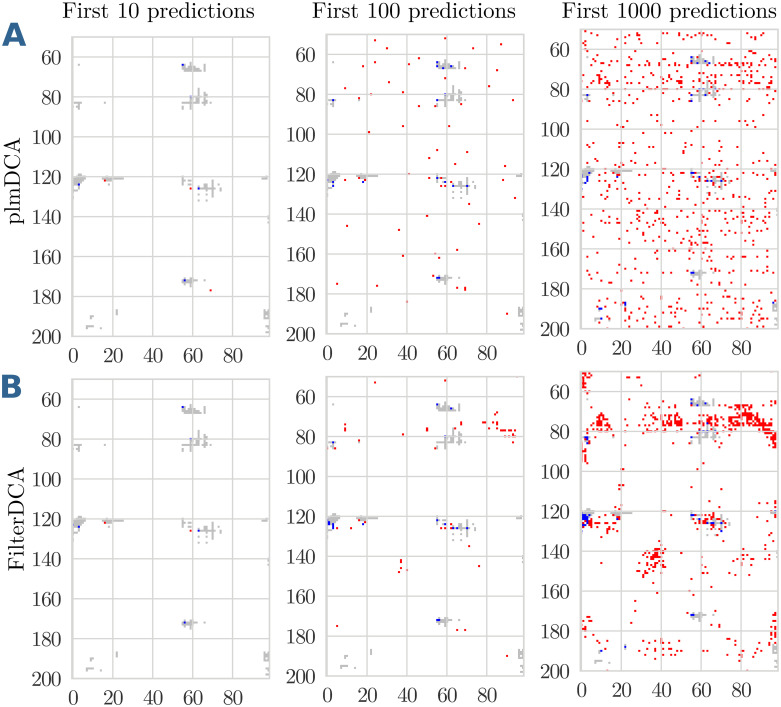
Contact map prediction for the interaction between the domain families PF01557 and PF09298 co-occurring in Fumarylacetoacetate (FAA) hydrolases. The upper panels (A) show the predictions of plmDCA, the lower panels (B) those of FilterDCA, for the first 10, 100 or 1000 predicted contacts. In each figure, the native contacts are shown in grey, TP predictions of the two methods in blue, FP predictions in red. This example shows a case where the plmDCA signal is weak, and consequently cleaning by FilterDCA does lead to a marginal improvement only.

Fig 7 show an unfavourable case. Already the first plmDCA predictions are of low accuracy and contain several FP. Also in this case, FilterDCA is able to remove many of the isolated FP of plmDCA, and it predicts clusters of contacts. However, due to the weak plmDCA signal, many of these clusters are collectively FP predictions. The final quality of FilterDCA depends essentially on the initial quality of plmDCA.

**Fig 8:**
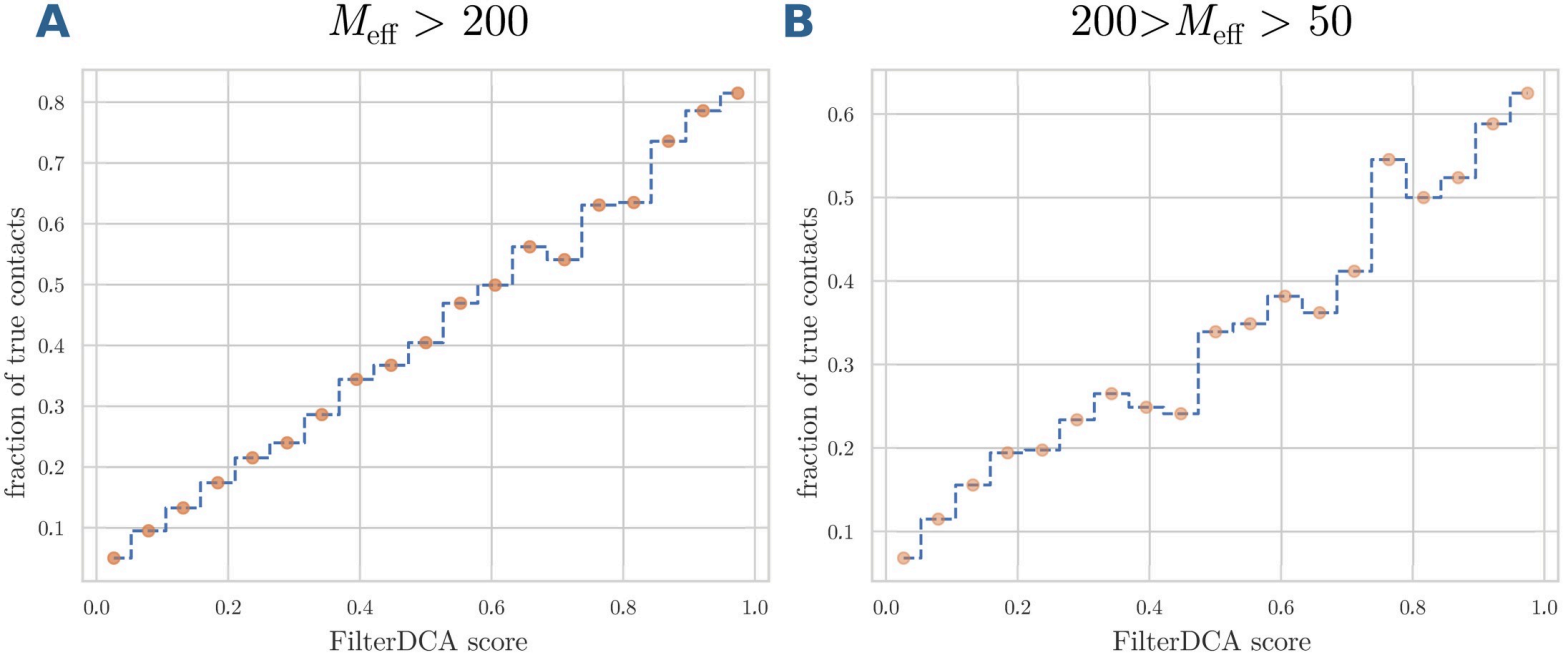
Fraction of true residue as a function of the FilterDCA score. The fraction of true contacts is evaluated in the test sets for large (panel A) and medium (panel B) MSA sizes, as a function of the predicted contact probability *P*(⊕|**x**) provided by FilterDCA (filter size 45 × 45). We observe a clear linear relationship, with a slope slightly below one due to overfitting effects resulting from the finite training set and the limited complexity of our regression model.

So far we have used the score of FilterDCA only for ranking residue pairs, with the expectation that high-ranking residue pairs are more likely to be in contact. However, the score is expected to provide *quantitative* information: for any two residues, the score *P*(⊕|**x**) predicts a probability of being in contact. In Fig 8, we compare this score to the fraction of true contacts between all residue pairs of given (binned) FilterDCA score within the test set. In the left panel Fig 8A, the result is shown for the class of large MSA. We find a very clear linear relation between predicted probability and actual contact fraction in the test set. The slope is slightly lower than one, which is to be expected from the simplicity of the logistic regression and the finiteness of the training set. However, about 80% of all residue pairs with a score *P*(⊕|**x**) > 0.9 are actually in contact, demonstrating that overfitting effects are rather weak. The situation is similar for the intermediate-size MSA depicted in Fig 8B. As to be expected, the results are more noisy and the slope is smaller than for the large MSA. We can therefore use the value *P*(⊕|**x**) of the best-ranking residue pairs to gain confidence—or not—on the interface prediction.

### Training on intra-protein / inter-domain contacts improves inter-protein contact prediction

In the introduction, we have motivated our work on domain-domain interactions and predicting inter-domain residue contacts by the idea that these interactions can actually serve as proxies for protein-protein interactions (PPI) and inter-protein contact prediction, but have more representatives in the structural databases, thus allowing for better structure-based supervision. Is this actually true?

**Fig 9:**
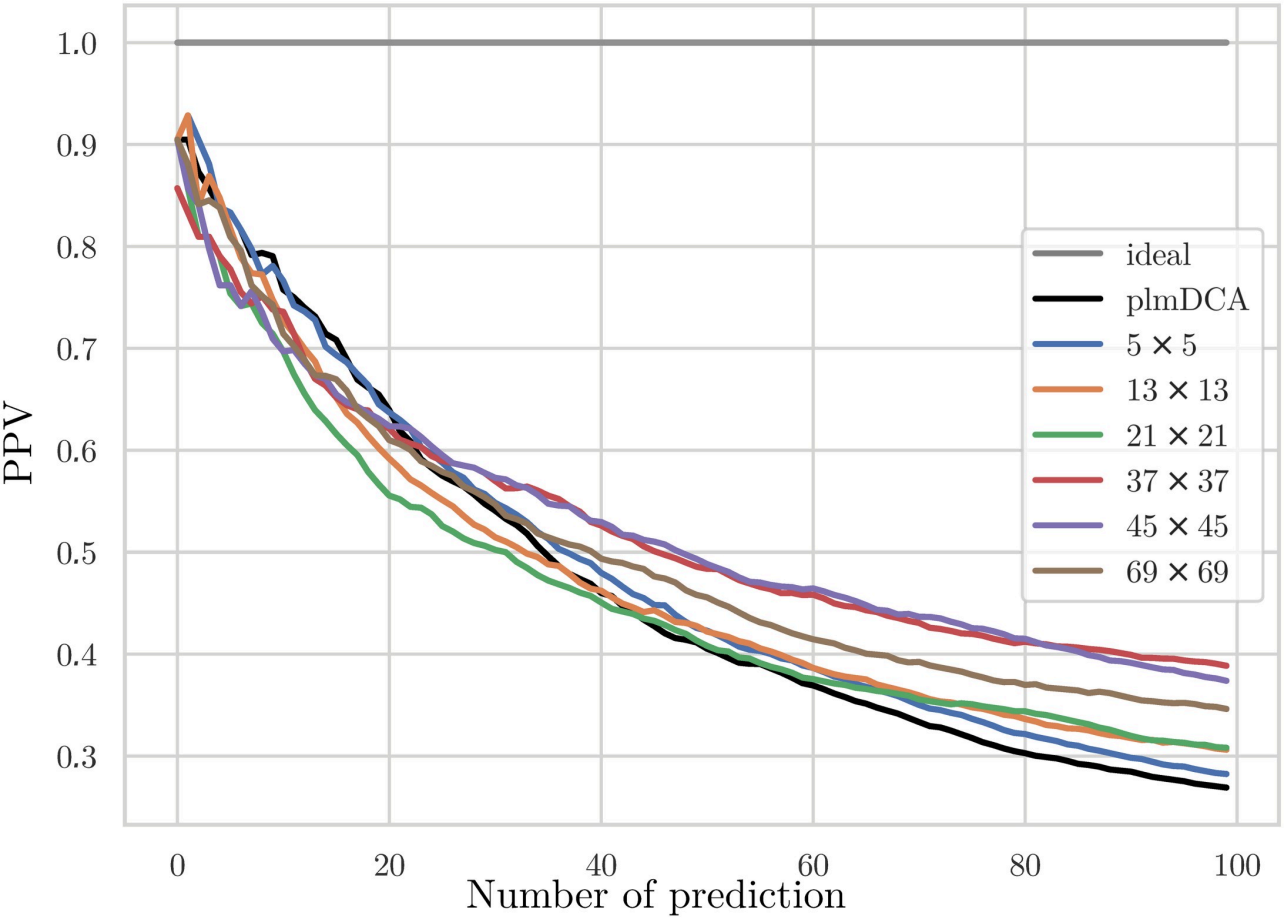
Positive predictive values of FilterDCA for inter-protein contact prediction. PPVs are shown as a function of the number of predictions, averaged over the different protein-protein interactions in the test set. Different filter sizes are compared to standard plmDCA.

To answer this question, we have used the PPI dataset of [21] as a test set, applying Filter DCA as learned on the intra-protein / inter-domain dataset used in the last section (case of large MSAs), for technical details cf. *Materials and Methods*. The results are depicted in Fig 9. We find that FilterDCA can be robustly transfered from inter-domain to inter-protein contacts, with comparable gains in PPV in the two cases (the general performance seems a bit higher, but we guess it is a direct effect of the smaller and curated PPI dataset in [21]). Interestingly, PconsC4 seems to suffer more from the transfer to PPI, the initially weak performance being even more pronounced than in the inter-domain case. This illustrates that the transfer itself is not trivial, with inter-domain contacts representing an intermediate case between intra-domain and inter-protein contacts.

### Comparing the performance of FilterDCA with deep-learning based contact prediction

It is instructive to compare the results of FilterDCA with some of the recent deep-learning based contact predictors. However, there are some subtleties:

- Most contact predictors are designed for intra-protein contacts, and an application to protein-protein interactions is not straightforward. As was shown by RaptorX-ComplexContact [16], a CNN trained on intra-protein contacts can predict inter-protein contacts, when a suitable concatenated MSA of the corresponding two protein families is supplied. We have therefore concentrated our comparison in the PPI case to three contact predictors:
  - RaptorX-ComplexContact is explicitely constructed for PPI and constructs its own MSA starting from two target sequences. The resulting dataset consequenctly differs from the MSA used for FilterDCA.
  - PconsC4 [20] and DeepMetaPSICOV [13] can be run on user-provided MSA. However, a subtlety has to be taken into account. The convolutional filters of the CNN underlying both approaches may overlap with both proteins, when applied to residues in the direct vicinity of the concatenation point of the MSA. This situation is not present in the CNN single-protein training set. As a consequence, we observe that this type of incoherent input inflates the numbers of false positives in both PconsC4 and DeepMetePSICOV. We therefore removed residues within 5 positions from the concatenation to enable a fair comparision.
  Methods requiring to provide a single target sequence are not directly applicable to the PPI problem and therefore remain unconsidered in our work.
- This is not a problem for domain-domain interactions, when both domains coexist in the same protein. However, we were able to verify the independence of our test set from the CNN training set only for PconsC4, therefore restricting the analysis to this method for domain-domain interactions.

**Fig 10:**
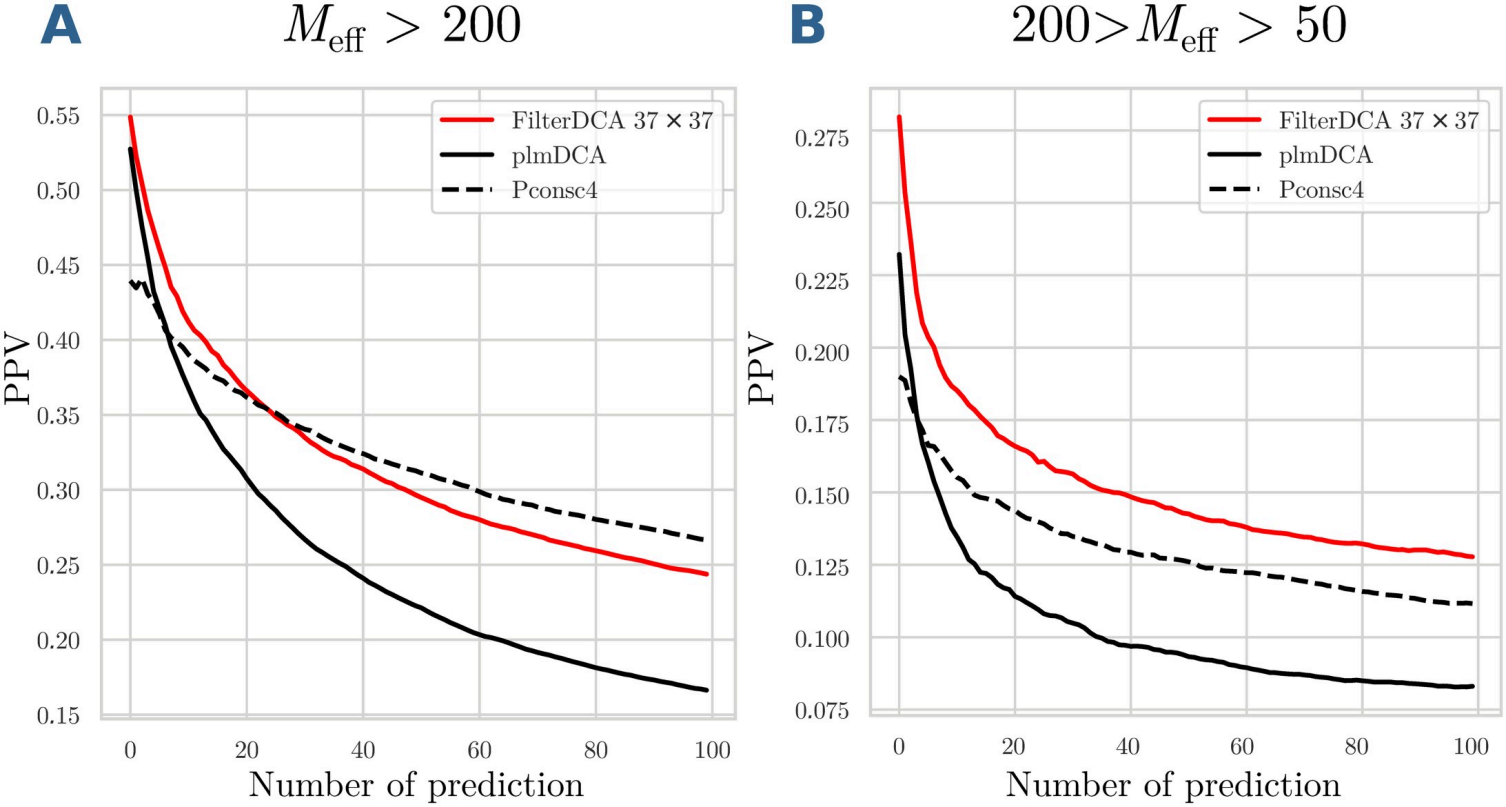
Positive predictive values for inter-domain contact prediction. PPVs for plmDCA, FilterDCA and PconsC4 are shown as a function of the number of predictions, averaged over the different domain-domain interactions in the test set. Predictions within 5 residues from the concatenation of the two domains are removed from the prediction, since they provide incoherent inputs to CNN and tend to produce many FP; this artifact strongly reduces the accuracy of CNN-based contact predictors.

The results for the latter case are shown in Fig 10. Interestingly, the performance of FilterDCA is comparable to the one of PconsC4. While initially being slightly worse due to some probably systematic mispredictions of PconsC4, the asymptotic behavior of PconsC4 is very similar to the one of FilterDCA, with a small advantage for PconsC4 in the case of large MSA, and for FilterDCA in the case of intermediate MSAs. However, on one hand it has to be noted that PconsC4 was trained on intra-domain contacts, and applied to inter-domain contacts, a fact which might systematically impact on the prediction quality of PconsC4. On the other hand, PconsC4 is trained on much more contacts than we need for FilterDCA.

**Fig 11:**
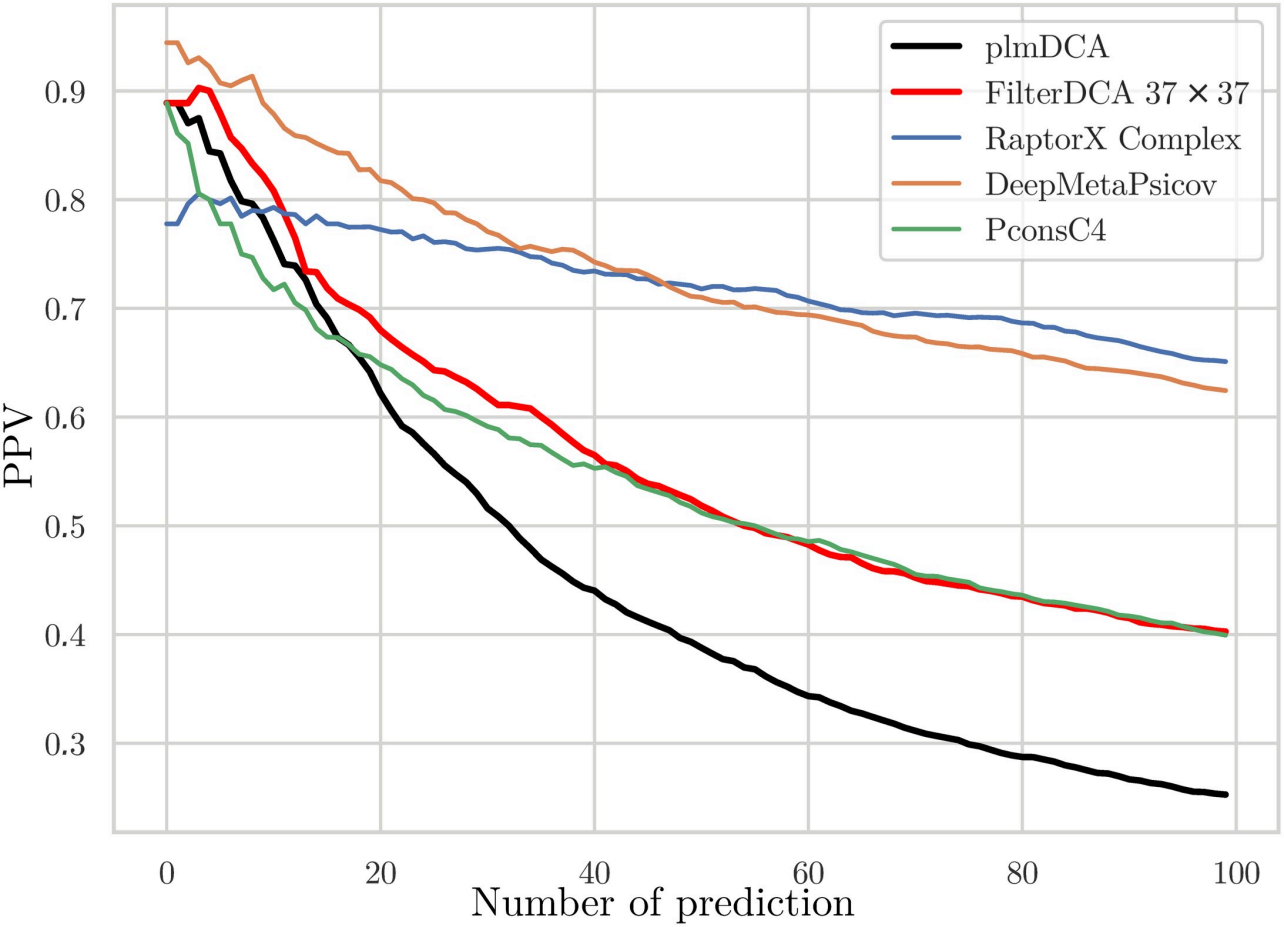
Positive predictive values for inter-protein contact prediction. PPVs for plmDCA, FilterDCA, PconsC4, RaptorX-ComplexContact and DeepMetaPSICOV are shown as a function of the number of predictions, averaged over the 18 different PPI in the test set, for which RaptorX successfully produced output. Predictions within 5 residues from the concatenation of the two domains are removed from the prediction, since they provide incoherent inputs to CNN and tend to produce many FP; this artifact strongly reduces the initial accuracy of CNN-based contact predictors.

As mentioned before, PPI allow for a comparison with two of the currently best contact predictors, RaptorX and DeepMetaPSICOV, in addition to PconsC4. The latter shows again a performance compatible to FilterDCA, while both RaptorX-ComplexContact and DeepMetaPSICOV have a coherently better performance, as is shown in Fig 11. Interestingly, with the exception of some initial difference, these two deep-learning-based methods have a very comparable performance, even if DeepMetaPSICOV was explicitly designed for intra-protein contacts.

## Conclusion

In this work, we have presented FilterDCA, a simple coevolution-based but structurally supervised contact prediction method. It is based on two different ingredients:

- Standard direct-coupling analysis DCA is used to detect coevolutionary couplings between pairs of residues from the sequence variability in sufficiently large MSA of homologous proteins. While strong couplings are known to accurately indicate residue-residue contacts, weaker couplings may not only result from coevolution, but also from the finiteness of the MSA used to learn the DCA model, and from biases in these data (e.g. phylogenetic correlations between homologous sequences). The sparsity of the strong couplings, as compared to the abundance of weaker couplings, limits the applicability of standard DCA for tertiary and quaternary protein structure prediction.
- Contact maps of proteins and their complexes are not random, but highly structured. Contacts tend to cluster together, and to show characteristic contact patterns depending in particular on secondary structure. These contact patterns are reflected by the patterns of coevolution in the vicinity of contacts.

FilterDCA uses simple supervised learning to identify residue pairs, which have relatively strong coevolutionary signals, but which are also coherent with the described contact patterns in a neighborhood of the residue pair under consideration. We have observed that this combination allows to improve the contact prediction when going beyond the cases of very strong individual DCA couplings, and to remove noise from the predicted contact maps.

The simplicity of our aproach makes it robust even for limited training sets (we have used domain-domain interactions), but leads itself to obvious limitations: building our supervision on explicitly constructed contact patterns, we cannot find alternative, unexpected and thus surprising informative features. More flexible architectures like convolutional neural networks may do so, and we actually find that two recent CNN-based predictors systematically outperform our simple approach in the case of protein-protein interaction, while a third method delivers comparable results.

It would now be interesting to see how far other biological features are informative about residue-residue contacts. At least three possibilities come directly to our mind: first, surface exposure may be a very interesting feature, in particular for protein-protein interactions between compact domains—only residues exposed at the surface of the monomers have a reasonable chance to form inter-protein contacts. Second, not all types of amino acids are biophysically compatible to form stabilising contacts. DCA couplings reflect such biophysical interactions, but a direct implementation of amino-acid interaction matrices might contribute to an improved contact prediction. Third, interaction interfaces form typically a limited number of connected patches on the protein surfaces—this may be used as a coherence measure between different predicted contacts. Each of these may give a contribution to contact prediction, leaving room for the future exploration of interpretable coevolution-based contact predictors.

Alternatively, we could try to use inspiration gained by FilterDCA to interprete the signal extracted by CNN-based contact prediction. In the very first layer, also CNN used filters of a mathematical form similar to the one we used. It might be interesting to look into the filters automatically learned from the training data, and to relate them to structural and physical-chemical properties of the proteins. Subsequent layers may combine these local features, to finally reach the observed accuracy in contact prediction. While the interpretation of the parameters of deep neural networks is recognized to be a typically very hard problem, the insight gained in our paper might help to unveil at least part of the success of CNN in the case of residue-residue contact prediction.

## Materials and methods

### Domain-domain interaction dataset

The basis of our dataset is given by the 3did database [22], which was constructed by selecting high-resolution PDB structures [18] containing multiple contacting Pfam domains [23]. We use the Aug 5, 2017 version which is based on Pfam v.30.0 and contains a total of 11.200 structurally-resolved domain-domain interactions. To get the joint MSAs we exclude homodimeric cases, and match sequences of domains co-localized on the same protein chain, i.e. we consider exclusively intra-protein inter-domain interactions. Finally, we map each residue in the MSAs to the corresponding positions in the PDB files. The mapping is done by aligning the PDB sequences to the profile HMMs of the PFAM domains through hmmalign [24], which allows to associate residue-residue distances to any pair of alignment columns.

Often only a part of the MSA can be mapped to the corresponding PDBs. We keep only MSA s with domain mapping coverage greater than or equal to 40% of the Pfam-domain length. The distance between two residues is defined as the minimal distance between all heavy-atoms of the two residues; in case the same residue-residue pair is associated with multiple PDBs, we assign the minimum distance between all possible copies. This assumes that any predicted contact (distance below 8Å), which is present in at least one PDB structure, is a true positive prediction. We further clean our dataset by requiring at least 10 and at most 2000 residue-residue interactions and removing few cases of coiled-coil structures which, due to repeated motifs, can lead to spurious coevolutionary signal.

At the end, we keep a total 2598 joint MSAs of pairs of contacting domains. A list of these domain-domain interactions is provided in the Supplementary data.

### Direct coupling analysis

We have run plmDCA [19] in A. Pagnani’s Julia implementation (available at https://github.com/pagnani/PlmDCA) with standard settings. Two outputs are of relevance: for each pair (*i*, *j*) of residues a DCA score *F*_*ij*_ is provided (via the statistically corrected Frobenius norm of the DCA coupling matrices), and the effective sequence number *M*_eff_. With the scope of predicting inter-domain contacts, we only consider residue position pairs with *i* in the first, and *j* in the second domain.

**Fig 12:**
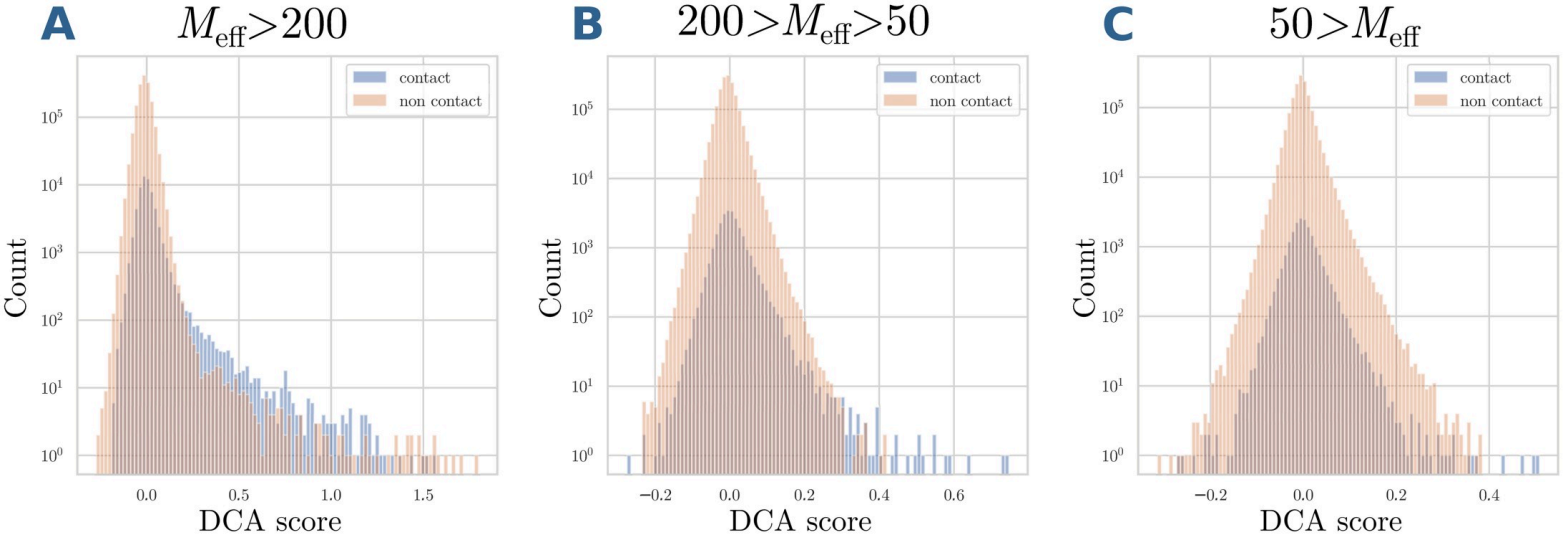
The distributions of DCA scores of contacts and non-contacts. For MSAs with *M*_eff_ > 200 (Panel A) and 50 < *M*_eff_ ≤ 200 (Panel B). Note that the enrichment of true positive predictions (contacts) is very high in the tail of large DCA scores. In fact, the majority of the pairs with DCA score larger than 0.3 corresponds to contacts. This is not the case for MSA with *M*_eff_ < 50 (Panel C), where the two distributions completely overlap.

It is well known that the accuracy of DCA predictions is strongly dependent on the number of sequences in the MSA or, more precisely, on the effective number of sequences *M*_eff_—the higher the better. DCA predictions are comparable only for MSAs having similar *M*_eff_. Thereby, we decide to split the 2598 MSAs of contacting domains in 3 datasets according to *M*_eff_, see Table 1, and to analyse them independently. As can be seen in Fig 12, which shows histograms of DCA scores for all contacting and non-contacting residue pairs, only for the largest *M*_eff_ a clear contact signal is contained in the largest DCA scores.

**Table 1:** We split the 2598 MSAs of interacting domains in 3 datasets according to the effective number of independent sequences, *M*_eff_. They contain approximately the same number of MSAs (first row) and inter-domain contacts (second row). In brackets, the percentage of inter-domain contacts is given with respect to the total number of inter-domain residue pairs.

	*M*_eff_ > 200	50 < *M*_eff_ ≤ 200	1 < *M*_eff_ ≤ 50
Num joint MSAs	842	758	998
Num contacts	274587 (1,7%)	193936 (1,3%)	204752 (1,1%)

### Filter score

With the aim of going beyond simple DCA predictions, we define a new score by applying structural filters on DCA predictions, cf. Fig 3. Let

$$\begin{array}{c} D_{ij}=(F_{i^{\prime }j^{\prime }}\;|\;i^{\prime }\in [i-\frac{k-1}{2},i+\frac{k-1}{2}];\;j^{\prime }\in [j-\frac{k-1}{2},j+\frac{k-1}{2}]) \end{array}$$ (3)

be the matrices of size *k* × *k* of inter-domain DCA scores, centered in residue pair (*i*, *j*) (i.e. windows of the full plmDCA output). We always choose *k* to be an odd number in order to get a square matrix centered around the central pair, in practice we use window sizes *k* between 5 and 69.

**Fig 13:**
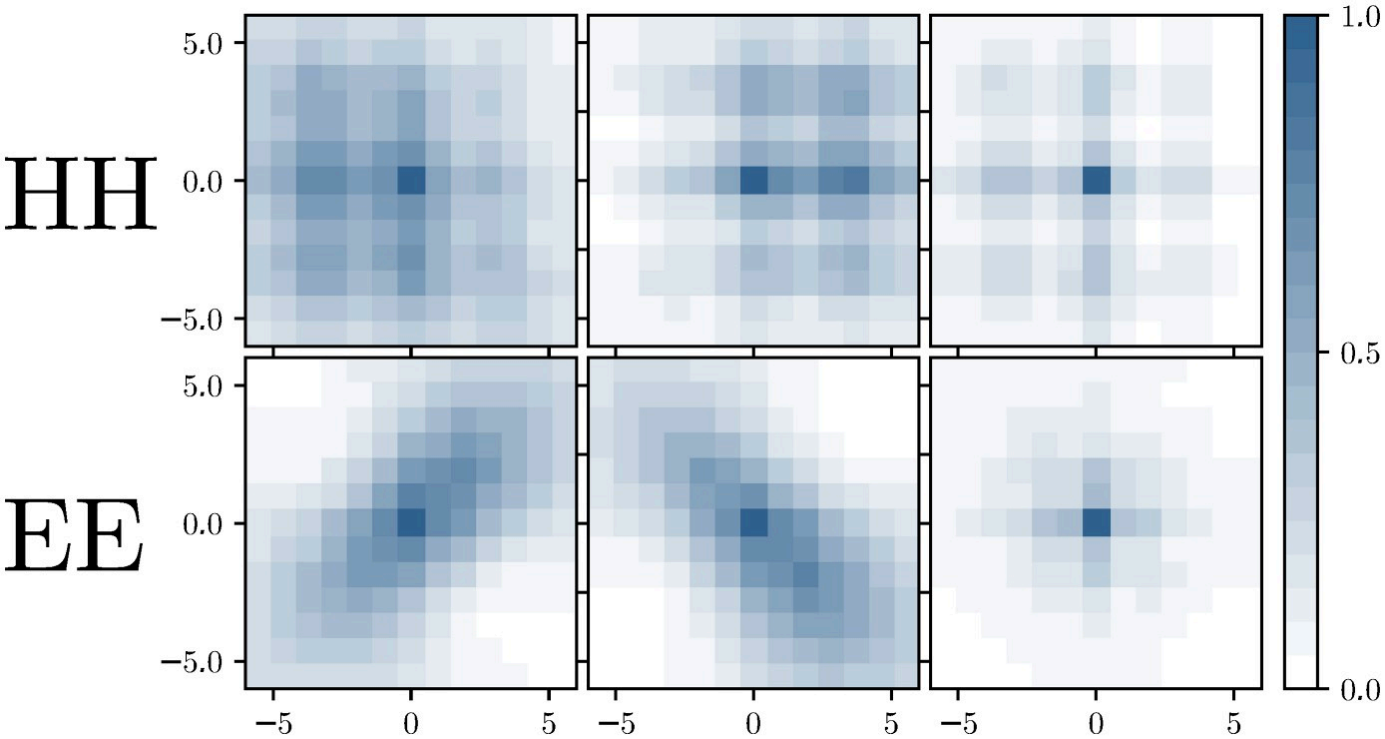
Filters used in the calculation of the filter score. The filters were determined from the inter-domain contact maps in the small MSA, with *k*-means clustering for *k* = 3 applied separately for HH and EE contacts.

The construction of the structural filters f∈S is as described in the main text: HH and EE inter-domain contacts are clustered into three clusters each, by using 3-means clustering. The filters are the centroids of the corresponding clusters. For all learning tasks (large and medium MSAs), we have used the same filters, determined using the contacts in the dataset of small MSAs, cf. Fig 13 for *k* = 13. Note that the filters are very similar to the ones in Fig 1, which were determined using all contacts in the large-MSA dataset. This illustrates the robustness of the filter construction.

For each of the 6 filters f∈S of size *k* × *k*, we compute the Pearson correlation between each *D*_*ij*_ and the filter. The central pair is removed from the calculation since, for the filters in S, it is a contact by construction and since the DCA score of the central pair (*i*, *j*) will be used directly. The new score, named *filter score*, equals the maximum of the 6 Pearson correlations.

A problem arises from pairs of residues closer than (*k* − 1)/2 to the border of the DCA matrix: the matrix *D*_*ij*_ is smaller than the filter matrix, and the procedure displayed in Fig 3 cannot be directly applied. In this case we compute the Pearson correlations only for pairs which are both contained in *D*_*ij*_ and in the filter matrix.

### Learning procedure

FilterDCA is based on supervised logistic regression which takes as input two features: (i) the standard DCA score, and (ii) the previously defined filter score, cf. Eq (1). It outputs the probability for a pair of residues to be in contact, as given in Eq (2). The bias *w*_0_ and the weights **w** = (*w*_1_, *w*_2_) are optimised using the ‘liblinear’ solver of the *sklearn* library [25].

Pairs of residues forming a contact are only a small fraction of all possible pairs, cf. Table 1). Thus, the training set is strongly imbalanced: the incidence of the *non-contact* class is dominant, being found in 99% of cases. We found that the performance of our classifier is improved if we restrict the training set to residue pairs with DCA score *F*_*ij*_ larger than zero, cf. Fig 12. In this case, the classifier concentrates on cases which show a more reliable coevolutionary signal, and which are concentrated closer to the decision boundary. Another further slight improvement has been achieved by scaling the filter scores, in both training and test set:

$$\begin{array}{c} x_{2}\to \frac{x_{2}-min\left(x_{2}\right)}{max\left(x_{2}\right)-min\left(x_{2}\right)} \end{array}$$ (4)

where *max*(**x**_**2**_) [*min*(**x**_**2**_)] is the maximum [minimum] filter score in the training set. While the classifier itself is invariant under this transformation, the *ℓ*_2_-regularisation used by *sklearn* is not, thereby influencing the final parameter values.

## Supporting information

S1 File List of domain-domain interactions. The file contains a list of all domain-domain interactions used in the manuscript, with Pfam IDs, PDBs and effective sequence numbers *M*_eff_.
(CSV)
